# A qualitative study of Telehealth patient information leaflets (TILs): are we giving patients enough information?

**DOI:** 10.1186/s12913-017-2257-5

**Published:** 2017-05-19

**Authors:** Reem Kayyali, Iman Hesso, Evelyn Ejiko, Shereen Nabhani Gebara

**Affiliations:** School of Life Sciences, Pharmacy and Chemistry, Kingston University London, Penrhyn Road, KT1 2EE Kingston upon Thames, UK

**Keywords:** Telehealth leaflets, Telehealth, Telehealth information, Discourse analysis, Patients perceptions, Telehealth guidance

## Abstract

**Background:**

The provision of patient information leaflets regarding telehealth has been perceived by potential consumers as a strategy to promote awareness and adoption of telehealth services. However, such leaflets need to be designed carefully if adoption and awareness among potential users is to be promoted. Therefore, the aims of this study were: first, to see how telehealth was portrayed in some of the existing telehealth leaflets (THLs). Second, to explore patients’ perceptions of the existing THLs and their engagement with the concept and how THLs can be optimised.

**Methods:**

A two-step approach was employed to address the aims of this study. The first phase involved the use of discourse analysis to compare 12 electronically and publically available THLs, with the existing THL guidance “Involve Yorkshire and Humber”. The second phase involved conducting 14 semi-structured interviews with potential telehealth users/patients to gauge their perception and engagement with the concept, using the two leaflets that were mostly matching with the guidance used. Six interviews were audio-recorded and eight had detailed jotted notes. The interviews were transcribed and thematically analysed to identify key themes.

**Results:**

The discourse analysis showed certain gaps and variations within the screened leaflets when addressing the following aspects: cost of the telehealth service, confidentiality, patients’ choices in addition to equipment use and technical support. Analysis of the interviews revealed patients’ need for having clear and sufficient information about the telehealth service within the THLs; in addition to, patients’ preference for the use of simpler terminologies for telehealth description and the provision of clear simple texts with pictorial presentations. The interviews also revealed certain limitations against adoption of telehealth by the participants, such as: lack of privacy and confidentiality of information, fear of technology breakdown and equipment failure, loss of face-to-face contact with healthcare professionals and being too dependent on the telehealth service.

**Conclusion:**

The current study showed a great variation among the screened THLs and highlighted certain gaps within the content and presentation of these leaflets. However, the study also highlighted certain key issues to be considered when designing THLs in the future to enhance telehealth uptake and use by patients.

**Electronic supplementary material:**

The online version of this article (doi:10.1186/s12913-017-2257-5) contains supplementary material, which is available to authorized users.

## Background

Telehealth has been increasingly advocated as an approach to support and assist in the diagnosis, monitoring, management and empowerment of patients with long-term complex health and social needs. It involves the use of information and communication technologies (ICTs) to allow the remote exchange of data (e.g.: blood glucose and blood pressure readings) and other information between healthcare professionals (HCPs) and patients [1, 2].

The Whole System Demonstrator (WSD) project is considered the world’s largest randomised controlled trial (RCT) of telehealth so far. In this trial, telehealth was found to significantly reduce hospital admission rates (*p* = 0.017), length of hospital stay (*p* = 0.023) and mortality rates (p <0.001) in the UK [3]. Despite such advocacy associated with the use of telehealth, its adoption is still poor in the UK [4] and other countries as well [1]. Evidence generated from survey studies in the literature highlighted some of the most frequent reasons behind non-participation in telehealth services among patients, these include: being too busy, preference for existing services, belief that technology could not help them as well as discomfort with technology [5–7]. The study of Sanders et al. [2] was one of the important studies which explored reasons for patients’ decline or withdrawal from the WSD trial. Lack of confidence with technology/requirements for technical competence and operation of equipment, expectations and experiences of disruption to the existing services, perceiving telehealth as a threat to identity, independence and self-care, and cost were the most identified barriers to participation and adoption of telehealth within the WSD trial among service users [2].

The provision of leaflets to patients is a requirement for effective self-management of health conditions in the current National Health Service (NHS) policy in England [6]. Providing leaflets for patients is a form of information. However, the knowledge and understanding gained from leaflets will not only depend on the facts included but also the discourse used in the leaflets [8]. Discourse cannot be separated from its social context [9] and this has consequences in relation to how language and discourse are viewed. An important feature to consider within the leaflets is the social influence produced by the reader’s interaction with the text via understanding and interpreting the terms and terminologies used; how people respond to texted informational materials depends considerably on how such information is designed [10].

The provision of patient information leaflets regarding telehealth has been perceived by potential consumers as a strategy to promote awareness and adoption of telehealth services [1]. However, such leaflets need to be designed carefully if adoption and awareness among potential users is to be promoted. Hence, the aims of this study were: first, to see how telehealth was portrayed in some of the existing/available telehealth leaflets (THLs). Second, to explore patients’ perceptions of the existing THLs and their engagement with the concept and how THLs can be optimised.

## Methods

A two-step approach was used to address the aims of this study. The first phase involved the use of discourse analysis to evaluate some of the existing THLs. The second phase involved conducting semi-structured interviews with potential telehealth users to gauge their perception and engagement with the concept.

### Phase one: discourse analysis and comparison of THLs with the available guidance

Discourse analysis was done on 12 electronically and publically available THLs that are developed by service providers of telehealth. To retrieve the leaflets, an online search was conducted using Google and Google scholar as search engines. Search terms used included: ‘telehealth information leaflets’ ,‘telehealth patient guide’, ‘telehealth patient information’, ‘telehealth patient leaflets’ or ‘telehealth leaflets’ The search resulted in the identification of 20 leaflets. The first 12 retrieved leaflets were selected and used, on the basis that these leaflets were more easily available/ retrieved by patients who want to seek information about telehealth over the internet. The remaining 8 leaflets identified could be mapped to existing leaflets; hence it was deemed that the first 12 identified leaflets (Table 1) provided saturation for the analysis to start. Most leaflets (*n* = 10) were provided by the NHS, however; this would have been expected given that the NHS is the main healthcare provider in the UK. Discourse analysis has been chosen to address the first aim of this study because it is a method where text is not merely viewed as a form of written words but as a form of social practice [8]. As the main barriers for telehealth adoption are related to social factors example: cost, confidentiality, technical skills and fear of losing healthcare professional-patient interaction [2, 5–7, 11, 12]; it was deemed essential to investigate how the leaflets addressed this from a discourse analysis viewpoint. Therefore, discourse analysis was used to investigate how language is represented and related with non-linguistic symbols such as pictures in THLs [13].

**Table 1:** The patient information leaflets on telehealth included in the study

Source/Leaflet	Leaflet title	Organisation
THL1	Supporting your independence and wellbeing with telehealth and telecare	NHS- 3million lives project
THL2	Telehealth- Information for patients and carers	NHS- Wandsworth Clinical Commissioning Group
THL3	Telehealth- ‘A new way to support your health care at home’.	NHS- Wales
THL4	Would you like to take control of your condition?	NHS- South West Yorkshire Partnership NHS Foundation Trust and Bassetlaw Clinical Commissioning Group
THL5	Telehealth Patient Information Leaflet.	NHS- Sunderland Teaching Primary Care Trust.
THL6	Telehealth in Bournemouth, Poole and Dorset.	NHS Bournemouth and Poole and NHS Dorset.
THL7	Telehealth- Supporting you to live more safely at home	Involve Yorkshire and Humber
THL8	Managing Long-Term Conditions- Patients Choice	NHS- East London NHS Foundation Trust
THL9	Telehealth monitoring	NHS- Bristol Community Health
	Patient guide	
THL10	The Long Term Conditions Psychological Support Service	NHS- Northamptonshire Healthcare NHS Foundation Trust
	Patient Information Leaflet	
THL11	The Human Touch	NHS- Kent Community Health NHS Trust.
	Transforming Community Services in Kent.	
THL12	Telehealth	Medvivo
	Aiding healthy independence	Integrating Health and Care

A prior online search was conducted at the beginning of the study to explore whether a THL guidance exists to help identify the themes for the discourse analysis. Search engines used included: Google and Google scholar. Search terms/phrases used included: telehealth leaflet guide/guidance, guidance for telehealth leaflets. The literature search identified a guidance for THLs produced by “Involve Yorkshire and Humber” (see Additional file 1), it was the only guidance for THLs identified at the time this search was conducted. The themes that were generated from the THL guidance reflected the current knowledge about telehealth adoption in the literature. The 12 leaflets that were afterwards identified as mentioned in the previous section were then compared to the themes generated from the THL guidance. Each leaflet was evaluated against the themes identified based on the guidance to look for similarities and differences across the collected leaflets. The two leaflets that reflected most of the themes were then used to gauge patients’ understanding and perceptions during the interviews in the second phase.

### Phase two: semi-structured interviews

A convenience and snowballing sampling strategy based on local knowledge and proximity to the researchers was used. This was adopted as a strategy to enable the feasibility and convenience of face-to-face interviews. Participants were approached in person by one researcher in three ﻿general practitioner (GP) surgeries located within Southwest London and were provided with written information sheet about the study to consider. Participants were included in the study if they were: over 18 years, suffering from long-term conditions (LTCs) and willing to participate. Snowballing, whereby participants were referring to friends or family members attending the GP surgeries was also employed to enhance recruitment [14]. Permission was granted from the GP surgeries to conduct the interviews at the premises, in private rooms to maintain confidentiality. Interviews were conducted with 14 patients; nine males and five females (Table 2). The interview schedule consisted of 10 open-ended questions (see Additional file 2) and was aided by two chosen sample THLs , to gauge patients perceptions about existing THLs, knowledge and understanding of telehealth gained from the two leaflets, engagement with the concept, perceived limitations, and how these leaflets can be further optimised. Sample size was based on data saturation, which was shown by some researchers to occur at the first 12 interviews [15]. In this study saturation occurred at the 11th interview, since no new data was emerging after the 11th interview. The stopping criterion for data saturation was three. Hence 14 interviews were conducted. The stopping criterion for data saturation is defined as the number of interviews that can be done without any new information, after which recruitment can be stopped [16]. However, all interviews were included for the analysis.

**Table 2:** Participants’ characteristics

Patients characteristics		Number of participants (out of *n* = 14)
Age group	21–30 years	4
	31–40 years	1
	41–50 years	1
	51–60 years	2
	61–70 years	2
	71–80 years	3
	81–90 years	1
Gender	Male	9
	Female	5
Diseases/Illnesses	Type 1 diabetes	3
	Type 2 diabetes	1
	Type 2 diabetes and hypertension	4
	Type 1 diabetes, high blood pressure and kidney transplant	1
	Asthma and other conditions	4
	COPD & hypertension	1

At the corresponding surgeries, the participants were first given 10–15 min to read the two sample THLs before the start of the interview. Written informed consent was obtained from participants prior the interviews. Six interviews were audio-recorded and eight had detailed jotted notes taken for those unwilling to be recorded. The interviews lasted an average of 20 min. Interviews that were not recorded took longer time (an average of 7 min more), as the researcher ensured to take sufficient detailed notes while conducting the interviews. Audio recordings and jotted notes were manually transcribed. Thematic analysis of the responses was done using inductive (from data) and deductive (from literature) approaches. The coding of data was guided by the following initial coding categories: perceived knowledge and understanding of telehealth based on the leaflets, additional benefits portrayed for telehealth, optimisation of THLs, perceived limitations to the use of telehealth, and preferred format of THLs.

The transcripts were read and re-read several times, coded manually and independently by two researchers. Thereafter, the codes were checked by a third researcher and discussed by all researchers to ensure consistency of findings.

### Ethical consideration

Ethical approval was granted for this study from the Research Ethics Committee at Kingston University London (ref: 1213/045).

## Results

### Discourse analysis and comparison with THL guidance

Although the Involve Yorkshire and Hamber guidance was useful, it did not set the criteria in a stepwise manner to enable the critique of THLs against it. After reading the guidance, the following criteria (Table 3) were identified and grouped into themes.

**Table 3:** Different aspects presented in the collected leaflets in comparison to the established guidance criteria

Theme	Guidance criteria	No of Leaflet(s) matching the criteria
• Knowledge	• What is telehealth?	• 8 (THL1, THL2, THL3, THL5, THL6, THL7, THL9, THL12)
	• How does telehealth work?	• 8 (THL1, THL2, THL3, THL5, THL6, THL7, THL9, THL12)
• Outcomes	• What will be done about the results?	• 6 (THL1, THL2, THL3, THL5, THL7, THL9)
• Reassurance	• Technology and equipment (ease of use): reassure patients that they do not need to be computer or technology experts.	• 7 (THL2, THL3, THL4, THL5, THL6, THL7, THL9)
	• Support regarding technical problems.	• 5 (THL2, THL3, THL6, THL9, THL12)
	• Confidentiality: state it directly and clarify who gets to see patients monitoring results	• 2 (THL3, THL5)
	• Costs: mention that the service is free for the patient.	• 7 (THL3, THL4, THL5, THL6, THL7, THL9, THL10)
• Benefits	• What are the benefits of telehealth?	• 9 (THL1, THL2, THL3, THL4, THL6, THL7, THL8, THL9, THL11)
• Choice	• State that the service is not a substitute for the traditional face-to-face consultations and patients still have the choice of seeing their HCPs when required. Telehealth is an extra service and not a substitute for emergency services.	• 6 (THL2, THL3, THL5, THL6, THL7, THL8)
• Previous Experience	• Patients positive quotes about telehealth These quotes provide a source of effective communication of patients’ good experience with telehealth services and consequently will encourage other potential service users.	• 4 (THL2, THL4, THL7, THL9)
• Further Information	• Who to contact for further information about the service in general: there will be somebody to help/ guide the user to understand e.g.: HCP or someone at end of phone.	• 10 (THL1, THL2, THL3, THL4, THL5, THL6, THL7, THL8 THL10, THL11)
	• Information availability in other languages/formats.	• 3 (THL2, THL5, THL7)
• Appearance	• Visual Presentation using pictures, not only words. Recommended: Variety of pictures to be included most importantly pictures of how the kit works; patients using the kits at home: images showing different ages, ethnicity and gender of people using the kit.	• 8 (THL2, THL3, THL5, THL6, THL7, THL8, THL9, THL12)

The 12 leaflets were then contrasted in relation to the themes identified to guide the discourse analysis.

Based on the available guidance, the required criteria that should be covered in a leaflet compared to that in the collected leaflets are shown in Table 3, in addition to the number and percentage of criteria met by each leaflet in Table 4.

**Table 4:** Number and percentage of criteria met by each of the analysed THLs

Leaflet	Number of criteria met by each leaflet (*n* = out of 13)	Percentage of criteria met by each leaflet
THL1	5	38%
THL2	11	85%
THL3	11	85%
THL4	5	38%
THL5	10	77%
THL6	9	69%
THL7	11	85%
THL8	4	31%
THL9	9	69%
THL10	2	15%
THL11	2	15%
THL12	4	31%

#### Depiction of telehealth service

Symbolisation of telehealth service varied considerably between the leaflets. Different terminologies were used to symbolise the service across the analysed THLs (Table 5). Despite that most leaflets used images in combination with text, yet not all leaflets provided images of the real technology/equipment to be used by itself except for THL2, THL3, THL5, THL7. Eight out of twelve leaflets described what telehealth was as a service (Table 3). Telehealth was the terminology used in all the leaflets. To aid the understanding of the concept, it was followed by a description of the benefits. The focus in these descriptions was on the main benefit of telehealth as a tool to improve or support patients’ health in their own homes. In addition, there was a variation in the description of how telehealth works across the eight leaflets. Some stated that telehealth works by providing a device depending on the patient’s condition. The recorded vital signs will either be sent automatically to a response/monitoring centre (THL2, THL7, THL9) where abnormal readings will be identified or entered into the device and communicated via a telephone line (THL3, THL5, THL6). Two leaflets (THL1, TH12) provided a flow chart of how telehealth works. THL4 was found to contain a very brief and basic description of telehealth procedure and hence could not match the criteria listed in the used guidance.

**Table 5:** Terminologies used to symbolise telehealth service in the analysed THLs

Portrayed description of the telehealth service	Leaflets
Small unit	THL1
New, user-friendly system	THL2
A home hub	THL3
A special piece of equipment	THL4
A small monitor	THL5
The homepod	THL6
A small pieces of equipment	THL7
Using a small hand held device	THL9
The Human Touch	THL11
Remote health monitoring	THL12

THL10 provided a description about the healthcare condition for which the telehealth service can be used, rather than the telehealth service itself. It was mentioned in the leaflet that the HCP will explain to the patient how the system works through an actual appointment, making the concept vague for potential users.

#### Outcome of using telehealth services

Only six leaflets emphasised the outcomes of using telehealth (Table 3). This was mainly by encouraging patients that it is not all about taking their vital signs but also about monitoring them remotely. Hence, patients will be contacted and action will be taken by clinicians as required in case of any discrepancies in readings.

*“Your readings are sent automatically to a response centre where any abnormal readings are identified. If this happens your GP or healthcare professional will be informed and make contact with you to take any necessary action.”* [THL2]

#### Reassurance about telehealth in the leaflets

A set of leaflets addressed some of the barriers that were repeatedly highlighted in the literature among previous telehealth users to affect adoption of telehealth such as cost, confidentiality, technology use and technical skills, disruption of established healthcare services [2, 5–7, 11, 12]. However, this varied considerably between the leaflets.

##### Technology and equipment (ease of use)

Seven leaflets made a point related to the technological aspect of the service by stating that the incorporated devices in the service were easy to use and install (Table 3), a point related to alleviating patients’ anxiety and fear when using a service incorporating technology, especially for elderly patients.

*“The equipment is easy to use and you will not need any specific technical skills to operate it.”* [THL6].

##### Support regarding technical issues/problems

Regarding support for technical difficulties, only five leaflets addressed the issue (Table 3), which is related to providing a sense of security to patients while using the service. Two leaflets (THL2, THL3) addressed this directly by providing specific telephone numbers to contact in case of technical problems or questions about telehealth equipment. Whereas in the other two leaflets (THL6, THL9), patients were clearly assured about the provision of clear contact information for any technical issues upon the installation of the service. Whereas in THL12, patients were provided with an online link to access in case of any technical problems.

##### Confidentiality

Only two leaflets (THL3, THL5) provided assurance regarding the confidentiality of the service in a clear way (Table 3). These leaflets stated directly that the information provided is confidential, highlighting that only members of the healthcare team in charge had access to the results and that information is sent through a secure network.

*“Your information is confidential and will only be seen by the telehealth monitoring team”* [THL5]

In another two leaflets, the phrase ‘confidential information’ (THL4) and ‘anonymous and confidential’ (THL10) was stated within the general description of the service without providing any further explanation. However, four of the other leaflets (THL1, THL2, THL6, THL9) did not address this issue clearly but indirectly by conveying the idea of secure data transfer within the context of the leaflet without further clarification. This means that the information will be translated and understood differently based on the information provided on the various THLs. Whereas, the remaining five leaflets did not provide any reassurance for patients’ confidentiality whether directly or indirectly.

##### Cost

Seven leaflets conveyed the idea that the service was free of charge (Table 3). Three leaflets (THL4, THL7, THL9) stated this clearly and directly (Table 6). Whereas, the remaining three leaflets (THL3, THL5, THL6) conveyed the idea indirectly; two leaflets (THL3, THL5) stated that the provided monitor will use an existing telephone line and the information is sent using a free-phone number without mentioning anything about the cost of the equipment, and the last leaflet (THL6) stated that the equipment will be provided free of charge without mentioning anything about call charges despite that the information is to be sent via telephone line (Table 6).

**Table 6:** Messages regarding the cost telehealth service in the analysed THLs

Portrayed message regarding the cost of the telehealth service	Leaflets
*“The information will be sent by a telephone to a secure computer… These telephone calls will not cost you anything as they use an 0800 freephone number.”*	THL3
*“The service is led by a dedicated team of nurses, it’s totally free ….”*	THL4
*“The monitor will use your existing telephone line and the information is sent using a free phone number so there will be no call charges.”*	THL5
*“The equipment will be provided free of charge… and will be maintained again free of charge”.*	THL6
*“It is free….”*	THL7, THL10
*Telehealth is part of the ongoing support you will receive from your healthcare team and there is no cost for using it.”*	THL9

#### Benefits of telehealth

Depiction of telehealth as an approach for managing patients with LTCs was predominant in all leaflets. In some leaflets, this was clearly conveyed in the title (THL8, THL10). However, titles varied considerably in the initial message conveyed. Some titles depicted telehealth as an approach to support self-independence and wellbeing, being a new way to support healthcare at home, taking control of patients’ condition, supporting patients to live more safely at home (Table 1). All these titles portrayed telehealth as a means to make patients take an active role in the management of their conditions, hence addressed a patient empowerment discourse.

In addition, benefits of telehealth service were featured in nine leaflets, either via a list or with a brief description, hence meeting the guidance criteria regarding benefit (Table 3). Some of the common benefits of telehealth suggested by the leaflets were: (1) added peace of mind, (2) greater understanding of your condition and increased confidence, (3) less risk of unplanned admissions to hospital, (4) greater freedom to get on with day-to-day life and (5) less anxiety for carers and family. Some leaflets (THL2, THL3, THL6, THL11) were discussing the benefits within the context of the local and national community to emphasise on these benefits as a way to encourage people to use the service.

*“Telehealth has helped thousands of patients with similar conditions across the UK and we believe this is a great step forward to help you stay at home, stay healthy and self-manage your condition so you have fewer unplanned hospital admissions”* [THL6]

Interestingly, one leaflet (THL11) has mentioned the benefits of telehealth with reference to the WSD trial results, since it has been developed by the NHS in Kent, which has been one of the three sites in England where the trial was implemented. In another leaflet (THL1), graphical drawings of 4 case scenarios were used to depict how telehealth can help people of different ages and conditions.

#### Patients’ choices in using telehealth

Telehealth was conveyed as an optional service in six leaflets only (Table 3). This means that patients can change their mind whenever they wish to withdraw from using the service and are still free to visit their usual HCP when needed. This also unpacks the anxiety associated with the introduction of new services or the fear of losing face-to-face contact with HCPs as a result of technology introduction into patients’ routine of life.

Two leaflets (THL3, THL7) conveyed this directly by clarifying to the readers that they are still free to visit their usual HCP when needed and highlighting that telehealth is not a replacement for emergency services; one of the leaflets (THL7) addressed the issue of choice as the first message. Another two leaflets (THL2, THL5) conveyed this by highlighting that patients should contact their HCP or emergency contact in case of feeling unwell. One leaflet (THL8) focused on highlighting that telehealth is not an emergency service without further reassurance about the choice of contacting the HCP when needed. Whereas, the remaining leaflet (THL6) addressed this issue by providing the patients with the choice to withdraw from using the service when they want, highlighting that telehealth is not a permanent arrangement, without mentioning that telehealth is not a substitute for the emergency services (Table 7). However, the remaining set of leaflets did not include information on patients’ choices in using telehealth.

**Table 7:** Messages regarding patients’ choice in the analysed THLs

Portrayed message addressing patients’ choice in the telehealth service	Leaflets
*“A telehealth system is not a substitute for making contact with a health care professional if someone feels unwell. Appropriate advice must be sought at the earliest opportunity”.*	THL2
*“It is important to remember that Telehealth does not replace emergency or normal GP services”.*	THL3
*“However, this does not give an immediate alert and you should call your usual emergency contact if you feel unwell.”*	THL5
*“You should be aware that this does not need to be a permanent arrangement and you are able to stop using the equipment when you feel the time is right. You can discuss this with your healthcare professional.”*	THL6
*’Telehealth is not an emergency service or a replacement for visiting health professionals”.*	THL7
*“Please remember that our telehealth service is not itself an emergency rather than it aims to help you understand how best to manage your condition”*	THL8

#### Patients’ positive experiences

Patients’ experiences were not integrated in eight leaflets. Four leaflets incorporated patients’ quotes from people who actually used telehealth, showing how confident they became with their health upon having a telehealth device to regularly monitor their condition (Table 3). Three leaflets; through providing more than one quote; focused on illustrating different benefits associated with telehealth use and providing testimonials of how people with different ages and conditions benefited from the use of telehealth. THL4 was the only one to mention the presence of short films on their website to show the stories of local people who have already used the service.

This aspect helps patients to shape their action regarding the use of telehealth using lay experience as a resource.

*“Telehealth has been great. I know when I’m becoming unwell and feel very supported”.* [Diabetes patient- THL2]

#### Contact for further information

Ten leaflets had contact details for further enquiries about the service in general, by including either telephone number, email or website to refer in case of any further questions (Table 3). There was no clear contact information in the remaining two leaflets (THL9, TH12). This message would encourage patients to seek additional information and clarifications, reveal any misunderstandings after reading the leaflets, which in return may help patients to make an informed choice about using telehealth.

Whereas, only three leaflets clearly provided contact details for requesting the available information in the leaflets in other languages and formats.

#### Appearance of the leaflets

Eight leaflets had visual presentation of images in combination with texts to depict telehealth as a service (Table 3) and were found to meet the criteria set in the guidance regarding appearance. However, there was no consistency in the types of pictures included across the eight leaflets. Some leaflets (THL2, THL7, THL8) were more elaborate with the use of pictures than the others (THL3, THL5, THL6, THL9, THL12). For example, THL2 had several pictures showing the kit by itself, happy patients beside positive testimonials about the service, a happy patient using the device, and a picture of a nurse standing next to a patient using the device. THL7 had a picture showing how the kit works, a picture of a happy patient with a positive testimonial about the service, and a picture of a happy patient using the kit. This provided a form of visual guidance to the functionality of the service. Whereas, THL3 for example, had one picture showing only the actual device itself without portraying any interaction between patients and HCPs.

The remaining four leaflets (THL1, THL4, THL10, THL11) did not meet the guidance criteria since they either had plain text messages without any pictures (THL11) or pictures that were not according to the specifications listed in the guidance (THL1, THL4, THL10). THL11 did not have any pictures or images and linked the description of the service with benefits of telehealth by describing it as a current service used by many patients who have reported an improvement in their overall confidence and added peace of mind. Whereas, in THL10, the only picture used was that of a normal telephone, as a symbol to indicate the use of telephone within the service.

Based on the guidance used in this study, the three leaflets: THL2, THL3 and THL7 met 11 (*n* = 11, 85%, Table 4 before) of the 13 listed criteria. However, for the current study it was deemed enough to use two leaflets for the second phase of the study in order to avoid overloading and confusing the patients with so much information and to facilitate the interview process with the patients in a timely manner, given that the interviews were conducted at GP surgeries. THL7 was not used in the second phase since it was produced by the Involve Yorkshire and Humber project, and therefore was based on that guidance. As the guidance was used to generate the themes for the discourse analysis, therefore the use of THL7 was deemed to may have limited the desired added themes/knowledge from the interviews. Therefore, the two leaflets THL2 and THL3 were chosen as sample leaflets and used during the semi-structured interviews, in order to see how THLs, especially leaflets that were not designed based on specific guidance, can be optimised from patients’ perspective.

### Semi-structured interviews

Analysis of patients’ interviews revealed the following five themes:

#### Telehealth perceived as a means for regular health monitoring/checks

Based on the understanding gained from the two leaflets, most of the interviewees were able to describe telehealth as a service where HCPs monitor patients’ conditions closely without the need of actual appointment to manage their conditions.

*“Enables doctors to monitor you with you just being at home …”* (Patient 1)

Interestingly, the perceived benefit of telehealth was also linked to monitoring by majority of the respondents, as indicated by the following phrases: *“monitoring results”, “healthcare monitoring”, “health monitoring”, “monitoring LTCs”, “supporting and monitoring health”, “patients’ monitoring at home”, “daily health monitoring”, “regular health check/monitoring”, “supporting patients’ health”.*

#### Simple alternative terminologies to telehealth

Most of the interviewees (*n* = 9) preferred to have simpler terminologies that they can easily understand. Participants tended to breakdown the term ‘*telehealth’* into simpler and familiar words when depicting alternative terminologies. Patients perceived two key elements as essential for simplifying telehealth: one was for the distance (remote/being at home) and the other was for the function of telehealth, which was mainly seen as monitoring. The following terminologies were proposed: “home monitoring”, “remote health monitoring”, “distance doctor”, “personal doctor”, “healthcare button”, “remote health management”, “healthcare at home” and “home health service”. Whereas, five respondents felt that telehealth was a good terminology and did not provide any suggestions for alternatives.

One of the respondents who provided the term “home monitoring” as alternative terminology, illustrated such a choice with the below quote:

*“To be honest, I don’t think many people understand the word telehealth, whereas if you use something that is more familiar, will actually make feel like they need it. For example, it’s an emergency, you know in the hospital when you feel it’s an emergency. So they may see it as home monitoring rather than what it really is, which to see from afar whether or not they are deteriorating”.* (Patient 1)

#### Essential information within THLs

Patients wanted to have brief explanation/stepwise guidance of how to use the monitoring system at home in a clear way with pictures. They also wanted THLs to include the experience of other people who have used telehealth, clarify that telehealth is a free service, provide supporting evidence that the service is actually working (e.g.: include statistics, percentages etc.…). They also wanted to clearly state the requirements needed to set up the service like broadband or existing telephone line and that support will be provided to them by professionals to use the required equipment.

*“ It should be made very clear that it’s free…..You know some people need very clear cut words to say it is actually free and for installation they are coming to do this and that, with clear instructions”.* (Patient 2)

*“Reassurance to patients that professionals will teach or instruct them on how to use the equipment”.* (Patient 4)

*“More description using simple words to aid understanding of readers with second language”.* (Patient 9)

#### Perceived limitations of telehealth

The most commonly associated barriers highlighted for not accepting telehealth were the lack of privacy and confidentiality of information, fear of technology breakdown and equipment failure, loss of face-to-face contact with HCPs and being too dependent on it.

*“Not sure where your information is going to and who would have access to them…”* (Patient 12)

*“Firstly, technology can breakdown….”* (Patient 3)

*“I suppose becoming too reliant on it, you know like every second you are not sure of something, may be your blood pressure you want to strap it around your arm. I don’t think this is healthy”* (Patient 6)

#### Textual and pictorial presentation preferred for THLs

The majority of respondents preferred having a combination of pictures and text messages. Patients expressed their preference to have a limited simple text and avoid technical terms. Participants stressed on the use of pictures to aid in illustration and perceived such combination to be essential because people understand things differently; some people are good readers, some like pictures and some people have English as a second language.

*“The design of the leaflet using both text and pictures would be a good idea because some people are good readers and some like pictures”.* (Patient 2)

*“Better with both pictures and texts.”* (Patient 4)

*“A combination of both pictures and texts is mostly preferred.”* (Patient 8)

## Discussion

Results generated from the interviews in this study provided an insight into how different potential users perceive telehealth as a concept and how they prefer such information to be designed and presented.

The interviews highlighted patients’ preference to have limited text that is simple and understandable with pictures and avoid technical jargons. Including pictures is a very important aspect in THLs. According to McGuire’s information processing theory which explains the communication/persuasion process of information, pictures play an important role in the persuasion process [17, 18]. Evidence in the literature highlights the importance of pictures to facilitate attention, understanding and remembrance of health educational materials especially for elderly and people with low literacy skills [18]. It is also important to include relevant pictures, since pictures unrelated to texts tend to have no beneficial effect on the understanding [18]. Interestingly, our interviewees were also able to identify some additional issues to optimise THLs beside the criteria set by the used guidance. Most of them wanted simpler terminologies for the term *‘telehealth’* that can be easily understood when reading the leaflets; they also wanted a clear and brief stepwise explanation of how the system works, and additional assurances regarding the actual functionality of the system and the needed requirements to set up the system.

Our discourse analysis showed certain gaps and variations within the screened leaflets. The study of Sanders et al [2] was found to provide a valid discussion platform with respect to the results of our study. The aforementioned study highlighted that concerns about cost were not addressed properly and this was among the reasons for some potential users to decline participation in the WSD trial. Interestingly, results generated from our interviews confirmed that users wanted this issue to be clarified in the leaflets. However, our discourse analysis showed that this issue was only addressed in half of the leaflets, highlighting the first gap with respect to the leaflets’ content.

Patients usually perceive telehealth interventions to be stressful and disruptive to existing management routines especially when they are satisfied with the current services offered to them and perceive such care to be simpler than the offered telehealth intervention [2]. This further emphasises the importance of addressing patients’ choice by stressing that the service is optional and clarifying that it is not a total substitution to traditional consultations when needed [19]. Patients in our study showed scepticism about the service in comparison to traditional consultations, particularly the fear of losing face-to-face contact with HCPs. However, only six leaflets addressed patients’ choice, suggesting a big gap in this area.

Patients in the current study expressed their preference to have experiences of previous users incorporated within THLs. Quoting patients’ positive experiences provides a source of sensory information that can reduce anxiety effectively in patients. In addition, sensory information can help the readers to increase their perceived involvement in the proposed intervention [20]. However, this was only featured in four leaflets.

With respect to patients’ reassurance about the ease of use of the devices, seven leaflets included this, suggesting another big gap. Tutty and O’Connor [20] emphasise that information given to patients should be reassuring and allay any fears they may have. Patients who withdrew or refused to participate in the WSD trial had misconceptions and misunderstandings that special skills were needed to operate the installed equipment [2]. Having a portable, easy, quick to use system has been identified to be an essential feature for the successful implementation of telehealth systems [11]. In a previous research, 12 out of 54 participants immediately withdrew from the study after experiencing difficulties in using the telemonitoring system [21]. Fear of technology breakdown and equipment failure were perceived as limitations among our interviewees as well. Participants in the current study also wanted to have evidence about the functionality of the service and a clear idea about the requirements needed to setup the service. Provision of support concerning technical difficulties in particular can alleviate some of these fears or misconceptions regarding technology. Nevertheless, our study showed that this aspect was only addressed in five leaflets.

Concerns about confidentiality have been previously reported as a barrier against telehealth adoption [1]. This was also perceived as a barrier to engage in the service among our interviewees. Our discourse analysis showed that confidentiality was clearly addressed in two leaflets only, which adds another big gap with respect to the content of THLs.

### Strengths and limitations

The study has several strengths and limitations. To our knowledge, this is the first study, which involves discourse analysis of THLs with the use of a THL guidance and semi-structured interviews with potential users to explore perceptions and preferences for optimising THLs. According to potential consumers, provision of THLs has been perceived as a strategy to promote awareness and adoption of telehealth services [1]. However, the results of the current study highlighted gaps in the content and presentation of THLs, which could provide an insight into why the adoption of telehealth is still poor in many countries, despite the widespread advocacy for its use. Limitations of the study also exist. No sufficient information was collected on the interviewees’ characteristics including ethnicity, race, class, etc. and this could have been useful to illustrate any differences in telehealth understanding. In addition, the study explored participants’ understanding of telehealth based on two sample THLs and it would have been ideal to explore people's knowledge of telehealth before they had read the leaflets. Eight interviews were detailed jotted and not audio-recorded due to participants’ preference, which might have an impact on the amount of data collected. However, it was noted that these interviews took a longer time which in return highlights the awareness of the researcher for this point and their caution to take sufficient information to address this issue. Moreover, not all interviews were audio-recorded, and one researcher has conducted the interviews. This in return might have an influence on the quality of the data collected, as there is no recording available to later confirm the handmade notes. Although data saturation was taken into consideration, yet the fact that not all interviews were audio-recorded, the use of convenience sampling and having a small sample size with all participants being from an urban community within Southwest London, may have an impact on the generalisability of the results.

## Conclusion

Our discourse analysis showed a great variation among the screened THLs. Certain gaps have been highlighted within these leaflets when compared against the themes derived from the THL guidance. Gaps pertaining to content were mainly: assurances regarding the technology involved and technical support, cost, confidentiality, patients’ choices. Results generated highlighted the need to consider simpler terminologies for the term telehealth. Written information should be clear and explicit, using simple text and pictorial presentation. Furthermore, it should incorporate patients’ experiences to highlight benefits associated with telehealth as a service. Providing sufficient information is key for engaging service users [1]. Users of telehealth need to be also assured about the functionality of the service they are going to use and the requirements needed to set it up. Failure to address these issues within THLs may contribute negatively to patients’/potential service users’ adoption and participation in telehealth services. Therefore, the above discussed points should be carefully considered when designing THLs in the future as a potential strategy to enhance telehealth uptake and use by patients.

## Additional file

Additional file 1: Appendix 1. Patient-focussed information about telehealth: Guidance for telehealth information materials for patients. (PDF 71 kb)
Additional file 2: Appendix 2. The interview schedule. (DOCX 15 kb)

## Abbreviations

GP: General practitioner
HCPs: Healthcare professionals
ICTs: Information and communication technologies
LTCs: Long-term conditions
NHS: National Health Service
RCT: Randomised controlled trial
THLs: Telehealth Leaflets
WSD: Whole System Demonstrator
