# Supplementary material for: A qualitative study of Telehealth patient information leaflets (TILs): are we giving patients enough information?
Source: BMC Health Serv Res. 2017 May 19;17:362. doi: 10.1186/s12913-017-2257-5 (PMC5438507; doi:10.1186/s12913-017-2257-5)
Supplement: Additional file 1: — Appendix 1. Patient-focussed information about telehealth: Guidance for telehealth information materials for patients. (PDF 71 kb) [file 12913_2017_2257_MOESM1_ESM.pdf]

involve
Yorkshire & Humber
leading a vibrant voluntary sector

Patient-focussed information about telehealth: Guidance for telehealth information materials for patients

Background

Involve Yorkshire & Humber worked with the Health Innovation and Education Cluster (HIEC) Yorkshire & Humber on a small project to co-produce an awareness raising leaflet about telehealth. Involve supports and promotes the role of the voluntary and community sector in Yorkshire and Humber.

The background to this project was that the Yorkshire & Humber Strategic Health Authority (SHA) wanted to improve awareness of telehealth by working with users and potential possible future users and their supporters to produce a patient/user focussed leaflet and to test drafts for clarity and usefulness.

The aim of the project was to work with focus groups of current or potential telehealth users to find out their views and preferences about awareness raising leaflets on telehealth and then compile a draft leaflet.

What we did

We researched the available literature for users of telehealth products, collecting actual examples from health bodies and commercial equipment suppliers.

We initially ran focus groups with telehealth users, or people who might become users and some health and social care professionals. We worked with a lead researcher in service user and carer involvement at the University of Leeds School of Healthcare

We also tested the sorts of messages that literature needed to embody, for example, the benefits of using telehealth and the reassurances users required, such as telehealth not being a substitute for face to face consultations with health professionals but an additional resource.

What should people know about telehealth?

- Some people are nervous of things that are different, so need to reassuring people of change
- Reassurance:
    - that you don't need to be a computer whizz & there will be support
    - that equipment can't be easily broken
    - worried it might go wrong
    - worried won't be able to use
    - need to know who will sort out any problems
- Cost – start the leaflet with "this is free to you as a patient" (or usually free)
- Who gets to see your results & what they do about them
- Need knowledge, e.g. if it gives blood pressure reading, what is normal for me? (in generic leaflet, could mention why it is important to monitor such information, or to ask your GP for more info, etc)
- Can still see your GP
- There will be someone there to help you understand, e.g. healthcare professional/ someone at end of phone

- Positive patient quotes
- Good experience
- Someone to help/guide you
- Reviews/comments
- Why is it going to help? What benefits do I get?
- Benefits:
  - usually free
  - easy to use
  - reassurance for user and family, can make you feel more secure
  - improve patient confidence. Can educated/inform you about your health
  - helps you to manage your health and stay at home
  - feel more in control when you know about your own health
  - live more comfortably at home
  - lets NHS professionals (regularly) check your results – without leaving your home
  - easier/ more convenient than going to you GP, for which you have to travel, book an appointment, etc
  - timely – when patients need it
  - one user felt too ill to be at home & was frightened with no immediate contact to doctors. Telehealth gave reassuring contact to health professionals
  - can (depending on system) give you feedback
  - help to give health professionals more regular information – helps highlight changes
  - if you're not feeling well, can check without having to go to the Doctor's
  - speed – can help quickly when something is wrong (although need to stress that may not be seen immediately/ not emergency service)
- How does it work, how do I use it?
- Include contact details & telephone number
- How information can be obtained in other formats/languages
- Choice: Is optional and can change your mind. Can still see your GP.
- Doesn't replace going to the Doctor's – it's an extra service

***Guidance on what makes a good leaflet*:**
- Visual. How does it grab you, would you pick it up? Font needs to be large enough to read
- Pictures & quotes from a range of patients so able to identify with other users
- Not too much information
- Whatever it is advertising needs to be available (SHA: want to create public demand for telehealth services)
- Address patient concerns
- Poster, leaflet or booklet? Possible need for an eye-catching poster, then a more detailed leaflet that can be picked up and read (especially to not isolate those without access to the Internet)
- It is important to show people at home using equipment and show the full equipment so potential patients know whether it takes up much space, etc
- Explain that telehealth is for anyone with a long term condition that needs monitoring, not just older people, and reinforce in visual images used
- Use larger font for visually impaired people
- Useful and patient friendly
- More specific information on the technology and service available in local areas could be included
- The more information available to prospective patients to "spread the news" the better
- Beneficial to have leaflets in surgeries, pharmacies, out patient departments, etc

Leaflet template

This leaflet shows the *types* of text, photos and information that people consulted think works best. These guidelines below explain the features that an effective leaflet needs to have –though local variations might use different photos or examples of actual kit.

Brief guidelines/top tips for telehealth leaflets aimed at patients

- Use plain English, rather than health ‘jargon’ or convoluted vocabulary
- Use photographs of a range of patients using the full kit in their home
- Use images to show different ages, ethnicity and gender of people using kit
- Use photos to demonstrate how kit works
- Show kit from more than one company/ for more than one condition
- Patient quotes of stories communicate the message effectively
- Make sure the typeface is not less than 14 points and do not put text over pictures, particularly white text on coloured backgrounds, for accessible reading
- Use a clean, uncluttered typeface such as Arial or Calibri
- Make sure there is clear information about the local telehealth system and who to contact for more information, ideally include a telephone number and other contact details
- Include reassurances about costs, benefits and how patients can continue to see their health professionals
- Disclaimer that it may not be monitored 24/7 and is not a substitute for the emergency services or visiting your doctor
- Don’t overload the leaflet with too much information and technical details – give basics and where to get more information from
- Offer different formats such as large print and different languages

Involve would like to thank the patients, telehealth users, carers and support staff whose contribution made this project possible.

Involve Yorkshire & Humber
Suite 4D Joseph’s Well
Hanover Walk
Leeds
LS3 1AB
t:0113 394 2300
f:0113 394 2301
e: office@involveyorkshirehumber.org.uk
w: www.involveyorkshirehumber.org.uk
