# Supplementary material for: A qualitative study of Telehealth patient information leaflets (TILs): are we giving patients enough information?
Source: BMC Health Serv Res. 2017 May 19;17:362. doi: 10.1186/s12913-017-2257-5 (PMC5438507; doi:10.1186/s12913-017-2257-5)
Supplement: Additional file 2: — Appendix 2. The interview schedule. (DOCX 15 kb) [file 12913_2017_2257_MOESM2_ESM.docx]

Additional file 2: The interview schedule for patients:

1. From what you have read from the leaflets, what does the term telehealth mean to you?
2. After reading the leaflets, what message do you think has been passed to you?
3. Apart from supporting you in your own home, reducing hospital admissions, what do you think is an additional message that telehealth can bring to you?
4. What is your preferred term for the concept of telehealth?
5. Based on the information you have read from the two leaflets; what benefit do you think telehealth would bring to your health?
6. From what you have seen and read from the two leaflets when describing telehealth, what representation would you have preferred in the patient information leaflet: text, pictures or a combination of both?
7. From reading the leaflets, what do you think would convince you to use telehealth as a service?
8. Based on the leaflets, what are your perceived limitations of telehealth as a service to you? Or What do you think would limit you from wanting to use telehealth as a service?
9. What else could we have incorporated or added to these leaflet(s) to improve it?
10. How would you describe the telehealth service to someone else?
